# Cure Kinetics and Inverse Analysis of Epoxy-Amine Based Adhesive Used for Fastening Systems

**DOI:** 10.3390/ma14143853

**Published:** 2021-07-09

**Authors:** Bilen Emek Abali, Michele Zecchini, Gilda Daissè, Ivana Czabany, Wolfgang Gindl-Altmutter, Roman Wan-Wendner

**Affiliations:** 1 Division of Applied Mechanics, Department of Materials Science and Engineering, Uppsala University, P.O. Box 534, SE-751 21 Uppsala, Sweden; 2 Christian Doppler Laboratory LiCRoFast, University of Natural Resources and Life Sciences, Peter-Jordan-Straße 82, 1190 Vienna, Austria; michele.zecchini@boku.ac.at (M.Z.); gilda.daisse@boku.ac.at (G.D.); roman.wanwendner@ugent.be (R.W.-W.); 3 Institute of Wood Technology and Renewable Materials, Department of Material Science and Process Engineering, BOKU University of Natural Resources and Life Sciences, Konrad-Lorenz-Straße 24, 3430 Tulln, Austria; ivana.czabany@boku.ac.at (I.C.); wolfgang.gindl-altmutter@boku.ac.at (W.G.-A.); 4 Magnel Laboratory, Department of Structural Engineering and Building Materials, Ghent University, Technologiepark-Zwijnaarde 60, 9052 Ghent, Belgium

**Keywords:** thermosets, curing kinetics, calorimetry, inverse analysis, material parameters

## Abstract

Thermosetting polymers are used in building materials, for example adhesives in fastening systems. They harden in environmental conditions with a daily temperature depending on the season and location. This curing process takes hours or even days effected by the relatively low ambient temperature necessary for a fast and complete curing. As material properties depend on the degree of cure, its accurate estimation is of paramount interest and the main objective in this work. Thus, we develop an approach for modeling the curing process for epoxy based thermosetting polymers. Specifically, we perform experiments and demonstrate an inverse analysis for determining parameters in the curing model. By using calorimetry measurements and implementing an inverse analysis algorithm by using open-source packages, we obtain 10 material parameters describing the curing process. We present the methodology for two commercial, epoxy based products, where a statistical analysis provides independence of material parameters leading to the conclusion that the material equation is adequately describing the material response.

## 1. Introduction

Especially in polymers, the material response is often nonlinear and challenging to model [1,2,3,4]. For thermosetting polymers, there are different phases in polymerization, which builds individual chains of covalent bonds by using subgroups leading to crosslinking. The curing process increases the stiffness known as hardening. Curing is an irreversible process such that the degree of cure monotonously increases and eventually stops, whenever the material is fully cured. Mostly, the material is used in the design assuming that this fully cured state is attained. For example, in electronics, integrated chip (IC) is held together in an epoxy die bonding and this thermosetting polymer is completely cured in a temperature chamber or under UV-light at the level of manufacturing. Within the chamber, the temperature is higher than the maximum possible glass transition temperature such that the fully cured material possesses highest stiffness possible. As curing is irreversible, in daily use, the degree of cure fails to alter such that the change of material properties may be justified by degradation but not post-curing. In the case of building materials like concrete [5] with fastening in the structural engineering, the fastening is attached mechanically or by using adhesion regarding a thermosetting polymer [6,7,8,9,10]. The hardening process continues in the ambient temperature at a lower temperature than foreseen for the maximum stiffness [11]. This fact often leads to a long curing process with a material response varying over days. The limitation is natural, because large geometries prevent building up a controlled environment for curing. Companies synthesize special products curing even at low temperatures relatively quickly with excellent adhesion properties. Therefore, modeling the curing process is of paramount importance especially in applications like post installed anchors, adhering layers in environmental conditions, and additive manufacturing with thermosets.

The curing process is measured by a scalar quantity called degree of cure from 0 to 1. Technically, this measure is the mass ratio of the solidified material in the bulk. For modeling the evolution of this degree of cure, there exist phenomenological governing equations with coefficients to determine from experimental results. Determination of coefficients is challenging in the case of evolution equations since they are differential equations to be solved [12], and thus, this inverse problem is highly nonlinear. Since the modeling approach is mostly phenomenological, there are different modeling approaches in the literature as well. As expected they all perform adequately even for the same set of experiments [13]. Often, simple evolution equations (modeling one catalysis) are used for estimating the cure kinetics and they deliver adequate results in complex settings [14]. We emphasize that in many engineering applications, curing is used for obtaining the stiffness of the hardened (solidified) material. Since the chosen curing model is compensated by the chosen relation between degree of cure and stiffness, it is challenging to quantify the feasibility of the used model for different settings. Hence, we directly model the curing process itself by using differential scanning calorimetry (DSC) measurements. The measurable quantities are heat flux and temperature, out of which it is possible to extract coefficients in an evolution equation. In the simplest case, the inverse problem may be simplified by using different methods such as a so-called isoconversional method [15,16]. Simplification is possible in specific types of evolution equations suggested in [17,18,19,20]. For more complicated models, simplification is tedious. Epoxy based thermosetting polymers have been analyzed in the literature [21,22,23,24,25]. We understand that curing kinetics with more than one amines and different catalysis processes lead to a complicated model. Thus, we want to solve the inverse problem by fitting the coefficients and solving the evolution equation in an iterative manner. Such a method lacks robustness since the fitting procedure may lead to coefficients creating convergence problems in solving evoluation equation numerically. We demonstrate a robust method circumventing this technical problem for a well-established evolution equation for thermosetting polymers. We apply the method and determine all material parameters of two epoxy based materials used as structural adhesives by means of DSC experiments.

During the curing of thermosets, occurring exothermal chemical process is of autocatalytic character. There are two common observations in this process. First, the temperature is coupled to the curing process. Second, the deformation fails to change the process. Hence, we assert that we model the phenomenon accurately by performing experiments, where we measure and control heat and temperature by ignoring mechanics. This approach is discussed by means of an adequate modeling for epoxy based materials in the next section. Such a model necessitates parameters to be determined by experiments. For an epoxy based material used commonly in building materials, experiments have been conducted by using standard calorimetry techniques called differential scanning calorimetry (DSC). These experiments lead to a dataset of heat and temperature. The data are generated into a useful form leading to material parameters by exploiting numerical techniques for inverse analysis [26,27,28]. We explain the experimental procedure as well as the inverse analysis in detail and demonstrate the usefulness of the suggested modeling approach.

## 2. Modeling

Curing hardens (solidifies) the resin material and we introduce as usual the degree of cure—also called conversion degree, ω, as a mass fraction (concentration) within a unit volume of the bulk (mixture of resin and solid). The degree of cure is of importance for a complete description [29,30,31,32,33] of the material and its computation [34,35,36,37]. We understand this degree of cure as a conversion degree from viscous resin, ω=0, to the solid thermoset, ω=1, such that material stiffness depends on this value (but not on its history) [38,39]. By starting with the partial mass balance for the solid and then assuming that no diffusion occurs, we end up with an evolution equation defining the rate of conversion degree, ω˙, based on the chemical reaction kinetics. This evolution equation allows us to calculate locally the degree of cure simply by integrating the rate over time, ω=∫ω˙dt. Therefore, the objective of this study relies on investigating an adequate evolution equation and determining its coefficients.

An evolution equation is developed based on the chemical reaction kinetics. For an epoxy material, mainly, there are two different catalysis causing the curing: autocatalysis and impurity catalysis. Reaction kinetics based on autocatalysis mechanism [40] is effected by hydroxy groups formed during catalysis. Hence, the following evolution equation [41,42] is suggested to be used

(1) $$\omega ˙=\left(k_{1}+k_{2}\omega^{m}\right){\left(1-\omega \right)}^{n}\;,\;\;k_{1}=A_{1}\exp\left(-\frac{E_{1}}{RT}\right)\;,\;\;k_{2}=A_{2}\exp\left(-\frac{E_{2}}{RT}\right)\;.$$

Coefficients for kinetic rates, $k_{1}$ and $k_{2}$, are modeled by $A_{1}$, $E_{1}$ and $A_{2}$, $E_{2}$, by using an Arrhenius type of relation with the gas constant R=8.314 J/(mol K) $\hat{=}$ G/K. Catalysis is modeled by the typical power law with *m* and *n* to be determined, we refer to ([43] Table 1) for a collection of material parameters. Especially for materials with a single state in networking and dominated by the autocatalysis, this model is sufficient. We will discuss two amendments in the following.

For a wide range of viscous fluids (here resin), decreasing the temperature leads to a power law divergence of viscosity [44] known as the glass transition temperature. The material goes from a rubbery state to a glassy state. Below and above glass transition temperature, $T_{g}$, different mechanisms take over in the curing process. This glass transition temperature is low for resin and it increases with curing. Consider an isothermal curing where the glass transition temperature increases eventually beyond the isotherm temperature used for curing. Below $T_{g}$, the glassy state leads to a chemistry steered slow curing. Mobility plays a dominant role in the rubbery state as molecules collide and form a network via crosslinking. Beyond a degree of cure, material vitrifies, as observed as $T_{g}$ increases beyond the isothermal curing temperature. Effected by vitrification, in this glassy state, the chemical rate is more dominant. In the rubbery state above $T_{g}$, mobility of polymer chains is high such that the curing process is fast. In order to introduce vitrification (transition to glassy state) dependent kinetics into the latter model in Equation (1), a possible approach as in [45] characterizes glassy and rubbery states occurring simultaneously. The idea has been introduced by [46] based on phenomenological observations combined with the free volume reduction during curing, which leads to a decrease in mobility. Another amendment is to include an analogous modeling for impurity catalysis as the specific epoxy is supposed to act differently regarding impurities. By following [47], we use only primary and secondary amines via autocatalysis and impurity catalysis (denoted by *c* in index) and collective kinetic rates, $K_{1}$, $K_{1c}$. These rates involve diffusion controlled mechanism, $K_{\mathrm{diff}}$, as well as chemically steered mechanism, $K_{1,\mathrm{chem}}$, $K_{1c,\mathrm{chem}}$, as suggested in [48]. Finally, we obtain the following evolution equation:

(2) $$\begin{array}{c} \omega ˙=K_{1}{\left(1-\omega \right)}^{2}\left(\omega +\frac{K_{1c}}{K_{1}}\right)\;,\;\;\frac{1}{K_{1}}=\frac{1}{K_{1,\mathrm{chem}}}+\frac{1}{K_{\mathrm{diff}}}\;,\;\;\frac{1}{K_{1c}}=\frac{1}{K_{1c,\mathrm{chem}}}+\frac{1}{K_{\mathrm{diff}}}\;,\\ K_{1,\mathrm{chem}}=A_{3}\exp\left(-\frac{E_{3}}{RT}\right)\;,\;\;K_{1c,\mathrm{chem}}=A_{4}\exp\left(-\frac{E_{4}}{RT}\right)\;,\\ K_{\mathrm{diff}}=k_{T_{g}}\exp\left(\frac{C_{1}\left(T-T_{g}\right)}{C_{2}+\left|T-T_{g}\right|}\right)\;. \end{array}$$

The diffusion rate, $K_{\mathrm{diff}}$, is modeled by the Williams–Landel–Ferry (WLF) equation in the rubbery regime and greater than the chemical rate, $K_{\mathrm{chem}}$, in several orders in magnitude. In the glassy state, the diffusion rate is nearly zero regarding the chemical rate. This interplay delivers a realistic prediction of conversion degree, ω, at low temperatures, where a so-called “partial freezing” inhibits to attain a complete curing, ω=1. Although the reaction never stops, as the rate is in several orders smaller, instead of days, the reaction takes decades to be completed. This model incorporates the glass transition temperature, which is often determined by a fit function [49] based on calorimetric measurements. For consistency, we follow [47] and use the relation based on [50,51], as follows:

(3) $$T_{g}=\exp\left(\frac{\left(1-\omega \right)\ln\left(T_{g,0}\right)+∆C\omega \ln\left(T_{g,\infty }\right)}{(1-\omega )+∆C\omega }\right)\;,$$

with $∆C=∆c_{\infty }/∆c_{0}$ as motivated by the ratio of the heat capacity change at the glass transition. We aim for determining the parameters $T_{g,0}$, $T_{g,\infty }$, ∆C as well as $C_{1}$, $C_{2}$, $k_{T_{g}}$, $A_{3}$, $A_{4}$, $E_{3}$, $E_{4}$ from calorimetry experiments.

## 3. Experiments

Two commercial epoxy based products, material A and B, have been used for all experimental studies in this work. The thermosetting polymer material is composed of two components: hardener and resin. Mixing both starts the curing process, immediately. Thus, the product is sold in a cartridge that separates the hardener from the resin and is designed to be pushed through a screwed-on static mixer tip with a pre-determined mixing ratio. Differential Scanning Calorimetry (DSC) has been used to register the evolution of the curing reaction using a Netzsch DSC F3 Maia (Netzsch Geraetebau GmbH, Selb, Germany). DSC is a well-established technique that allows to study the thermal response of a sample by controlling and recording temperature as well as heat flux of a small cell containing both a sample and a reference. The temperature is controlled, either kept constant (isothermal test) or varied linearly in time (dynamic test). We start with the balance of energy, consider the case that the deformation is neglected, and furthermore assume that energy is related linearly to the temperature, *T*, by using the specific heat capacity, *c*. Under these assumptions, the balance of energy reads

(4) $$c\frac{\partial T}{\partial t}+\frac{Q}{m}=H_{\mathrm{ref}}\omega ˙\;,$$

where *Q* denotes the heat flux measured in the DSC for a material of mass *m* resulting in an enthalpy (total energy for complete curing), $H_{\mathrm{ref}}$. In the case of an isothermal test, temperature is held constant such that the first term vanishes. Therefore, the measured heat flux—this power is tantamount to the supplied energy rate necessary for controlling the temperature fixed—is to be used for obtaining evolution of degree of cure, ω˙, as an experimentally obtained quantity.

### 3.1. DSC Measurements

The sample was an irregular shape of a small amount of material of weight 10.0 ± 0.1 mg injected directly inside an aluminum crucible. The reference is an empty sealed aluminum crucible of the same size. Both crucibles were clamped during the test. In order to improve the quality of the measurement, the cartridges of material were stored at low temperatures (5 °C), slowing down the first part of the reaction. After the weighing, the sample was immediately placed inside the machine and the measurement started as soon as possible to minimize any loss of data regarding the beginning of the reaction. The temperature was then increased rapidly (by approximately 20 °C/min) to the given isothermal temperature and kept constant until the curing reaction stops releasing heat. Four different temperatures were tested (30, 40, 60, and 80 °C) with a testing time going from 60 min (80 °C) to 1200 min (30 °C).

A subsequent dynamic test was performed to the same specimen after the isothermal test in order to obtain the attained curing degree. The temperature was initially decreased to 10 °C using liquid nitrogen. The DSC chamber was then heated up to 160 °C with a heat rate of 10 °C/min registering the heat released from the material. The same process was repeated for a second time in order to obtain a reliable baseline for the last measurement. Since the specimen was fully cured within the first temperature ramp, the baseline corresponded to a temperature increase by means of the specific heat of the material, first term in Equation (4). The difference between two temperature ramp tests denoted the energy released because of curing that denotes the missing energy for a complete curing, ω=1. For gaining access to the degree of cure during the corresponding isothermal DSC test, the obtained energy was normalized by the enthalpy of the complete reaction. This enthalpy was determined in analogous manner. For freshly mixed resin, the temperature was increased linearly from 5 to 160 °C with a heating rate of 10 °C/min in order to ensure that the curing reaction would have been completed. The sample was then cooled down to the initial temperature and the test was repeated to obtain a baseline, then the process was repeated a third time for ensuring that no post-curing phenomena did take place. The total amount of energy was used as $H_{\mathrm{ref}}$ in Equation (4).

The second DSC test performed on the material was a ramp test to obtain the glass transition temperature, $T_{g}$, for different conversion degrees. The sample for the DSC test was obtained by breaking the solid specimens in the middle, by taking some material from the middle part, and by grinding it into a fine homogeneous powder. The powder was placed in an aluminum crucible, weighed and clamped, then it was placed in the DSC together with a second crucible, the empty reference sample. The temperature of the cell was increased linearly from 0 to 160 °C two times per each sample to create a measurement and its baseline. The second measurement did not register any further reaction indicating that the curing process was already completed after the first run.

### 3.2. Experimental Data

**Figure 1:**
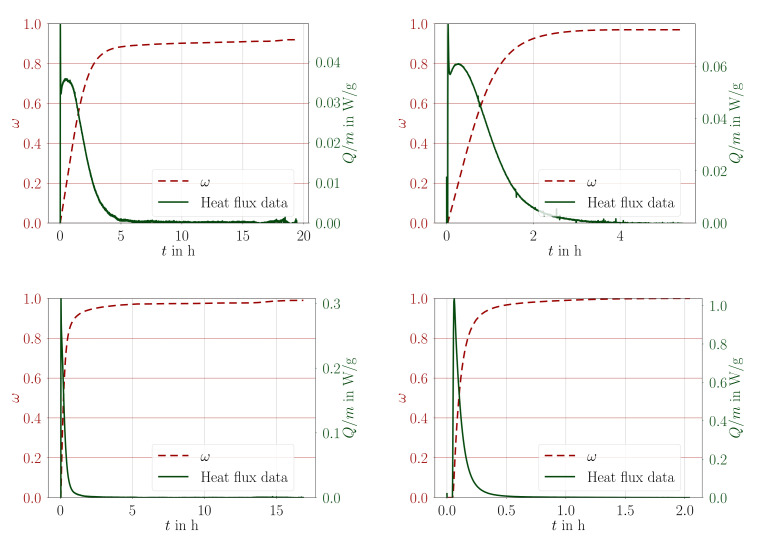
Experimental measurable heat flux (dashed, green line) and derived degree of cure (continuous, brown line) obtained during curing at 30, 40, 60, and 80 °C isothermal conditions, for the material material A.

The output of an isothermal DSC test at a given temperature is the specific heat flux, Q/m in W/g, as in Equation (4) released by the sample. This output is a function in time for one sample of unknown degree of cure. Subsequent dynamic test is used to obtain the degree of cure for this sample. Hence, we integrate Q/m function in time and obtain ω=ω(t) after normalizing by the obtained degree of cure. Heat flux and hence the evolution of conversion as obtained from experimental results are to be depicted in Figure 1 for the material A.

We repeat that there has been some seconds between pouring and initiating the measurement. Since the chemical reaction starts immediately, there is a peak in the heat flux indicating that the calorimeter’s feedback controller tries to regulate the heat flux resulting in a peak as well as negative values that is neglected in the postprocessing. The heat flux data is converted to the degree of cure by integrating numerically.

Obviously, the given temperature is strongly influencing the total duration of the exothermal reaction from roughly 1 to 10 h. The (dynamic) ramp tests performed after the isothermal measurements has been used for determining the degree of cure at the end of the isothermal test. In order to obtain this value, two subsequent ramp tests are postprocessed by taking their difference and integrating in time. The resulting specific energy (energy per mass) has been normalized using the enthalpy, $H_{\mathrm{ref}}$, necessary for the complete curing. Furthermore, this difference between dynamics tests has been analyzed for obtaining the transition from glassy to rubbery state. This glass transition temperature, $T_{g}$, is denoted by the position of the inflection point.

For defining $T_{g}$, aforementioned calorimetry experiments were conducted for a relatively small volume of material. In order to examine the scalability, we did perform additional experiments on the so-called dog-bone specimens. They were produced in agreement with DIN EN ISO 527-2:2016-06. The casting of the specimens was done utilizing silicon molds, in which the mortar was injected directly from the cartridge. Five different curing and post-curing protocols were generated in order to obtain five curing degrees, corresponding to different in situ conditions. After the treatment, the specimens were vacuum-sealed in plastic bags and placed in a fridge with a temperature of 6 °C in order to minimize the aging and post-curing effects. After one week of storage, the specimens were ground by a resin-bonded diamond grinding disc (Struers, MD-Piano series) at 300 rpm on a rotating plate of a grinding machine (Struers, Planopol-3) to obtain the final thickness of 3.8 ± 0.05 mm. The curing and post-curing protocols with their corresponding $T_{g}$ values are compiled in Table 1.

## 4. Inverse Analysis

All preprocessing, optimization, and postprocessing have been implemented in Python by using SciPy, NumPy, and MatPlotLIB modules. Two sets of different types of optimization procedures have been implemented, both based on scipy.optimize module. The first inverse problem is finding out 3 unknowns, $T_{g,0}$, $T_{g,\infty }$, ∆C, given in Equation (3). These parameters need to be obtained from Table 1 by minimizing the sum of squared error between experimental and fit values. Since unknowns are in a multiplicative way, there is no unique solution to this least squares optimization problem. Standard nonlinear regression solution techniques are implemented based on the Levenberg–Marquardt algorithm [52] with a linear loss as cost function. In order to detect the global minimum, several different starting values are selected in a stochastic manner from a reasonable but sizeable range.

For fitting Equation (3), unknowns $T_{g,0}$, $T_{g,\infty }$, and ∆C, need to be determined. The unknown $T_{g,0}$, represents the glass transition temperature of the resin. We emphasize that a glass transition of a resin is more a numerical parameter than a physical one. In addition to this fact, values from Table 1 are insufficient for its determination. We expect to have $T_{g,0}$ around 10 °C, therefore, we propose an additional iterative procedure in order to determine the best value. A small number of additional isothermal tests at 10°C are obtained directly in DSC. Then by setting $T_{g,0}\in \left[6;14\right]\;$°C and selecting randomly initial values for $T_{g,\infty }\in \left[50;100\right]\;$°C and ∆C∈[0;1], we obtained values for unknowns in Equation (3).

For determining $C_{1}$, $C_{2}$, $k_{T_{g}}$, $A_{3}$, $A_{4}$, $E_{3}$, $E_{4}$ from calorimetry experiments, we have implemented a nonlinear regression solving the ordinary differential equations for each iteration. The solution is handled by fourth order Runge–Kutta method. It is fast and accurate, since we simply solve the differential equation depending on time, as long as the parameters are physical. However, in this constellation, the gradient regarding unknowns might suggest unrealistic values leading to convergence problems in the solution of the differential equation. Therefore, we have exploited a trust region algorithm as in [53] with bounds solving sparse Jacobian [54] by using Cauchy loss as cost function with the aid of a SciPy implementation based on [55]. With this choice, by generating the exact Jacobian, we find the solution of the minimization problem of sum of squared error. We stress that all data is used for training and cost calculation. In order to increase the likelihood that the solution is the global minimum, we have chosen the initial values randomly from a given range with upper and lower bounds. We emphasize that the accuracy of the procedure is related to these bounds; we compile the utilized bounds in Table 2.

## 5. Results and Discussion

The material model used herein is motivated in a phenomenological manner. Therefore, we examine its validity by posing the question, if the number of material parameters is the minimum possible. In other words, we find out if this model is the simplest possible to accurately predict the materials behavior. We start the analysis with the material A, and use 3 experiments for 30, 60, and 80 °C as well as 2 experiments for 40 °C leading to in total 3×2×3×3=54 variations of experimental data. Each variation has been used to determine another set of parameters and this so-called bootstrapping method delivers that the parameters have no correlation with each other. We stress that an adequate material model necessitates parameters being non-correlated. In this way, we assess that the proposed model is the simplest possible material model. Parameters are determined by the aforementioned optimization problem, which is obviously nonlinear with many solutions. The bootstrapping method provides different results, there are at least four local minima suggested as possible solutions. Since there is no correlation, the parameters of different local minima are inadequate to compare. Instead we compare their accuracy to the experimental results and choose the solution with the smallest error, called the global minimum. For capturing the global minimum, we have used 50 times stochastic choice of starting points and repeated the procedure to assure the feasibility.

Material parameter $T_{g,0}$ may be understood as a glass transition temperature of the resin; however, from a technical point of view, the resin fails to have rubbery and glassy regimes. Hence, we need to develop a method for determining this parameter. First, by using an iterative process for obtaining the best $T_{g,0}$, we obtain all material parameters and then simulate with these parameters the additional isothermal tests at 10°C, see Table 3. Second, by simply summing up the error—absolute difference between measurement and simulation result—we observe that the best value is 9°C.

This best value is used in the fitting procedure in the case of isothermal calorimetry measurements. All data is used simultaneously such that the same parameter set simulates all results. These material parameters are compiled in Table 4 for both materials.

**Figure 2:**
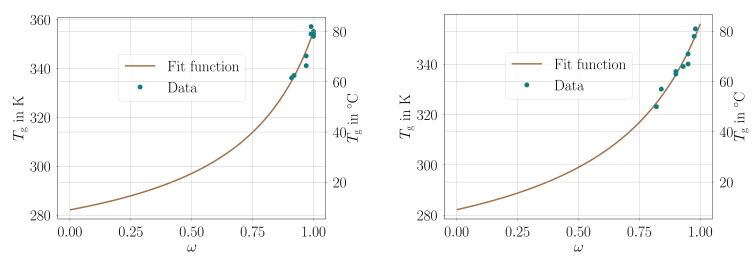
Comparison of the parameter fit resulted continuous line and experimental values as dots from Table 1. **Left**: material A. **Right**: material B. Parameters are in Table 4.

Since in general we cannot guarantee the global minimum of the inverse problem, the adequate judgment of accuracy is a comparison with the experimental results from Table 1 as seen in Figure 2.

**Figure 3:**
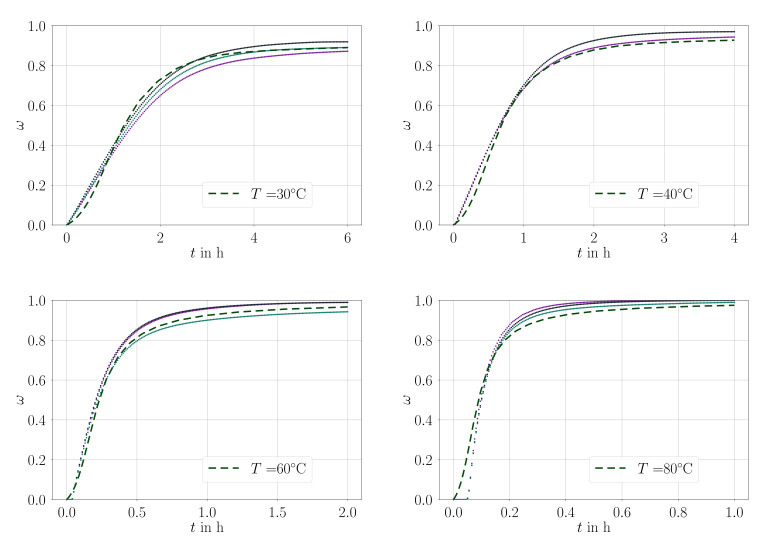
Comparison of the parameter fit (dashed line) with all experimental values (dots) used for the fit, material: material A.

**Figure 4:**
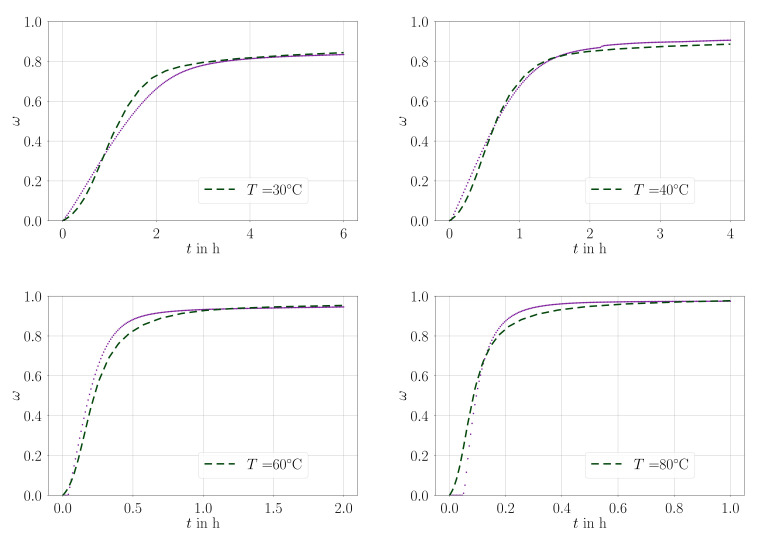
Comparison of the parameter fit (dashed line) with all experimental values (dots) used for the fit, material: material B.

Isothermal measurements for material A is demonstrated in Figure 3 and for material B in Figure 4 with parameters compiled in Table 4.

Materials A and B are both expected to be superior in quick hardening even in low temperatures; however, a few parameters show a stark difference. We stress that the combination of the parameters is of importance, although they are uncorrelated, varying one parameter fails to alter the response in any intuitive way, since the material model is highly nonlinear. As seen in Equation (2), the evolution depends on the current degree of cure explicitly in a nonlinear fashion, as well as indirectly as parameters depend on the glass transition temperature that depends on the degree of cure as given by the nonlinear relation in Equation (3).

As the parameters are uncorrelated, each of them is necessary and the model cannot be reduced furthermore. Yet their significance in the overall result differ. In order to demonstrate their sensitivity, at least for an arbitrary value in time, we use material A and simulate for 10 h at 20°C leading to 0.87 degree of cure as a reference value. By varying the parameters ±10%, we obtain the sensitivity of parameters as seen in Table 5.

We emphasize that this sensitivity analysis is a rough indication of importance of each parameter for detecting a value in time. These values will change significantly by choosing another time position as well as using non-isothermal conditions.

The material model uses temperature dependency but not temperature rate. This simplification is justified by near equilibrium thermodynamical processes. We emphasize that the curing phenomenon is seen as a “slow” process from a thermodynamics perspective. We have chosen an interval of temperatures related to engineering applications. Of course, outside of this interval, the material response may deviate. In practical applications, because of its exothermal character, temperature increases well above 30 °C but remains lower than 80 °C. Therefore, we expect to find a wide range of applicability with these parameters.

In real-life scenarios, non-isothermal conditions are a must and parameters demonstrated herein are valid in such cases. This fact is obvious since the optimization algorithm fits all isothermal measurements at once with the same set of parameters. Thus, the same model is expected to be capable of estimating the degree of cure even in situations, where the temperature changes. In order to validate this claim, we use Table 1 as test cases. We stress that these values are not used for determining the parameters such that we use them as a validation case. With both materials, by using parameters in Table 4, simulation results are compared to experimental results in Table 1. The results in Table 6 show an adequate accuracy. We stress that the experimental error is not known in these measurements.

## 6. Conclusions

Two commercial products, epoxy based thermosetting polymers, have been analyzed for their material response during hardening. Curing is an irreversible, chemical process including different types of polymerization leading to a hardened material measured by degree of cure. The cure kinetics depend on the material, and we have discussed for similar products—both materials are used as adhesives in fastening systems—the same model taken from the literature. Thermosets have a glass transition temperature indicating the change between rubbery and glassy phases. This change is reversible. Curing process is an irreversible process. We have taken these assumptions in the formal analysis:

- The glass transition temperature depends on the degree of cure. In other words, the history is irrelevant, current value of the degree of cure is of importance.
- The cure kinetics are modeled by an evolution equation. Neighboring particles fail to affect the kinetics, there is no flux in this model, justified by chemical reactions occurring locally.

Glass transition temperature dependency on the degree of cure is nonlinear and the evolution equation is a first order differential equation. This work has demonstrated a successful methodology to determine all material parameters in these equations. By using an inverse analysis implemented in Python language with open-source packages, we have determined all parameters and validated them by using additional experiments. We have observed and concluded:

- The simplest model is the proposed one with 10 material parameters.
- Isothermal tests in a DSC are adequate to determine the all material parameters necessary to describe the curing kinetics.
- Nonlinear regression problem by solving a differential equation is challenging yet possible by using a trust region algorithm.
- Non-isothermal conditions are captured equally accurately showing that the proposed model is a valid material equation.

An evolution equation is important for simulations [56] leading to predictive results in real-life applications, which is left to further research.

## Figures and Tables

**Table 1:** Curing and post-curing protocols for the definition of the five curing degrees for materials A and B.

Material	Degree of Cure, ω	Glass Transition Temperature, $T_{g}$	Curing
A	0.910	63 °C	30 °C/20 h
	0.920	64 °C	30 °C/20 h
	0.920	64 °C	30 °C/18 h
	0.970	68 °C	40 °C/5.5 h
	0.970	72 °C	40 °C/18 h
	0.990	81 °C	60 °C/2.25 h
	0.990	81 °C	60 °C/2.25 h
	0.990	84 °C	60 °C/17 h
	1.000	82 °C	80 °C/1 h
	1.000	81 °C	80 °C/1 h
	1.000	80 °C	80 °C/2 h
B	0.820	50 °C	23 °C/15 h
	0.840	57 °C	23 °C/24 h + 23 °C/24 h
	0.900	64 °C	30 °C/20 h
	0.900	63 °C	23 °C/24 h + 33 °C/24 h
	0.930	66 °C	40 °C/17 h
	0.950	71 °C	23 °C/24 h + 43 °C/24 h
	0.950	67 °C	60 °C/2.2 h
	0.975	78 °C	80 °C/1 h
	0.980	81 °C	23 °C/24 h + 110 °C/24 h

**Table 2:** Bounds used for parameter fitting in the least square error minimization.

	$A_{3}$ in 1/s	$A_{4}$ in 1/s	$E_{3}$ in G	$E_{4}$ in G	$k_{\mathrm{Tg}}$ in G	$C_{1}$	$C_{2}$
Lower bounds	1	1	100	100	10	10	10
Upper bound	$10^{5}$	$10^{5}$	$10^{5}$	$10^{5}$	$10^{3}$	$10^{2}$	$10^{2}$

**Table 3:** Determination of $T_{g,0}$ by an iterative process.

at 10°C	10 min	90 min	150 min	300 min	
	Degree of cure, ω				Error
Experiment	0.010	0.180	0.250	0.400	
$T_{g,0}=6\;{}^{\circ}$C	0.006	0.093	0.212	0.551	0.280
$T_{g,0}=7\;{}^{\circ}$C	0.006	0.096	0.222	0.567	0.282
$T_{g,0}=8\;{}^{\circ}$C	0.005	0.086	0.209	0.571	0.310
$T_{g,0}=9\;{}^{\circ}$C	0.007	0.103	0.227	0.559	0.263
$T_{g,0}=10\;{}^{\circ}$C	0.007	0.101	0.225	0.561	0.269
$T_{g,0}=11\;{}^{\circ}$C	0.006	0.092	0.214	0.556	0.285
$T_{g,0}=12\;{}^{\circ}$C	0.006	0.098	0.228	0.576	0.284
$T_{g,0}=13\;{}^{\circ}$C	0.008	0.127	0.273	0.604	0.282
$T_{g,0}=14\;{}^{\circ}$C	0.008	0.127	0.273	0.604	0.282
$T_{g,0}=15\;{}^{\circ}$C	0.006	0.097	0.222	0.562	0.277

**Table 4:** Determined measurement procedure and material parameters as a result of the optimization problem for materials A and B.

Material A			Material B		
**Variable**	**Value**	**Unit**	**Variable**	**Value**	**Unit**
$T_{g,0}=$	9	°C	$T_{g,0}=$	9	°C
$T_{g,\infty }=$	82	°C	$T_{g,\infty }=$	83	°C
∆C=	0.288	-	∆C=	0.332	-
$A_{3}=$	76,747	1/s	$A_{3}=$	96,748	1/s
$A_{4}=$	7500	1/s	$A_{4}=$	11,012	1/s
$E_{3}=$	45,966	G	$E_{3}=$	46,440	G
$E_{4}=$	48,319	G	$E_{4}=$	49,462	G
$k_{Tg}=$	740	G	$k_{Tg}=$	10.3	G
$C_{1}=$	47.3	-	$C_{1}=$	17.3	-
$C_{2}=$	57.9	-	$C_{2}=$	12.3	-

**Table 5:** Sensitivity analysis by varying the parameters.

	Degree of Cure			Relative Change	
Variable	Ref	+10%	−10%	+10%	−10%
$T_{g,0}$	0.87	0.70	0.93	−19%	+6%
$T_{g,\infty }$	0.87	0.84	0.91	−3%	+4%
∆C	0.87	0.87	0.87	0%	0%
$A_{3}$	0.87	0.88	0.87	1%	−1%
$A_{4}$	0.87	0.87	0.87	0%	0%
$E_{3}$	0.87	0.64	0.91	−26%	+4%
$E_{4}$	0.87	0.85	0.90	−3%	+4%
$k_{Tg}$	0.87	0.88	0.86	+1%	−1%
$C_{1}$	0.87	0.79	0.91	−9%	+5%
$C_{2}$	0.87	0.86	0.90	−2%	+3%

**Table 6:** Validation of the determined parameters for materials A and B by simulating curing protocols in Table 1.

Material	Curing	Experimental	Simulation	Relative Error
	30 °C/20 h	0.910	0.918	0.9%
	30 °C/18 h	0.920	0.916	0.4%
	40 °C/5.5 h	0.970	0.936	3.7%
	40 °C/18 h	0.970	0.954	1.7%
A	60 °C/2.25 h	0.990	0.971	2.0%
	60 °C/17 h	0.990	0.994	0.4%
	80 °C/1 h	1.000	0.975	2.5%
	80 °C/2 h	1.000	0.988	1.2%
	23 °C/15 h	0.820	0.848	3.3%
	23 °C/24 h + 23 °C/24 h	0.840	0.903	7.0%
	30 °C/20 h	0.900	0.897	0.3%
	23 °C/24 h + 33 °C/24 h	0.900	0.924	2.6%
B	40 °C/17 h	0.930	0.930	0.1%
	23 °C/24 h + 43 °C/24 h	0.950	0.949	0.1%
	60 °C/2.2 h	0.950	0.955	0.5%
	80 °C/1 h	0.975	0.977	0.2%
	23 °C/24 h + 110 °C/24 h	0.980	1.000	2.0%

## Data Availability

Code for the inverse analysis will be provided by the corresponding author on reasonable request.
